# Health literacy training program for community healthcare providers using hybrid online team-based learning in Taiwan

**DOI:** 10.1186/s12909-022-03646-7

**Published:** 2022-07-27

**Authors:** Jyh-Gang Hsieh, Jui-Hung Yu, Ying-Wei Wang, Mi-Hsiu Wei, Mei-Chuan Chang, Chao-Chun Wu, Shu-Li Chia

**Affiliations:** 1 Department of Family Medicine, Hualien Tzu Chi Hospital, Hualien City, Taiwan; 2 Department of Medical Humanities, School of Medicine, Tzu Chi University, Hualien City, Taiwan; 3 Department of Public Health, Tzu Chi University, Hualien City, Taiwan; 4 Department of Communication Studies, Tzu Chi University, Hualien City, Taiwan; 5 Department of Nursing, College of Medicine, Tzu Chi University, Hualien City, Taiwan; 6 Health Promotion Administration, Ministry of Health and Welfare, Taipei, Taiwan

**Keywords:** Hybrid, Online, Team-based learning, Community health providers, Health literacy, Learning experiences

## Abstract

**Background:**

Health literacy (HL) has proven to be a determining factor influencing the health of individuals. Community health providers (CHPs) work on the front line of improving public HL. Increasing their understanding of HL and their ability to incorporate HL into healthcare can reduce obstacles in healthcare services. This study evaluated the effectiveness of an HL training program for CHP by using the hybrid online team-based learning (TBL) model.

**Methods:**

A quasi-experimental study and focused group interviews were conducted. We developed a six weeks HL online course for CHPs. The program included teaching videos for pre-class preparation, a 90-min online TBL model, and a case discussion in the last two weeks. Team application activities were designed for each class to enhance knowledge application. A total of 81 CHPs from 20 public health centers took the course and provided complete data for analysis. Learning effectiveness was evaluated based on the familiarity, attitude, and confidence in implementing HL practices, course satisfaction, and participants’ learning experiences.

**Results:**

The comparison showed that the participants’ familiarity with HL (4.29 ± 1.76 vs 6.92 ± 1.52, *p* < .001), attitude (7.39 ± 1.88 vs 8.10 ± 1.44, *p* = .004), and confidence in implementing HL practices (6.22 ± 1.48 vs 7.61 ± 1.34, *p* < .001) increased after the course. The average satisfaction with the teaching strategies was 4.06 ± .53 points, the average helpfulness to practice was 4.13 ± .55 points, and the overall feedback on satisfaction with learning was 4.06 ± .58 points (the full score was 5 points). According to the learning experience of the 20 participants in the focus group discussion, the experiences of teaching strategies and the learning experiences of the HL course were summed up into two categories, seven themes, and 13 subthemes. The results showed a positive experience with the hybrid online TBL program.

**Conclusion:**

The use of hybrid online TBL model is a feasible and valid approach for the HL training of CHPs. The result can serve as a reference for the on-the-job training of various healthcare workers.

**Supplementary Information:**

The online version contains supplementary material available at 10.1186/s12909-022-03646-7.

## Background

Health literacy (HL) is defined as “The individuals’ capacity to obtain, process and understand basic health information and services needed to make appropriate health decisions” [1] Low levels of HL are associated with several adverse health outcomes and pose a significant challenge to public health [2]. Healthcare providers should be aware of the patients’ difficulties with low HL, communicate in plain language, and provide written materials that are easy to understand, enhancing HL and mitigating adverse health outcomes [3].

Recent studies have shown that Healthcare providers did not sufficiently understand the HL concept [4, 5]. They did not have clarity on applying HL strategies and evaluating patients with low levels of HL. The written educational materials were not applied effectively in practice, and they held negative attitudes toward the practice of HL-related care [6–9]. Therefore, there is an urgent need for HL continuing education among Healthcare providers.

Coleman et al. defined HL competencies for Healthcare providers as the knowledge, skills, and attitudes that health professionals must possess. Effectively incorporating HL in health services is an important step in the HL training of health professionals [10]. Providing information that is easy to understand and useful with appropriate communication skills is one of the major aspects of HL competencies for health professionals, and may prevent individuals from receiving services due to HL limitations [4, 10–13]. Many teaching methods have been applied to HL training courses to achieve this goal. Aside from the conventional lectures, standardized patients, interactive videos, and practical exercises are also widely used to promote the learning of HL skills [4, 12–15].

Team-based learning (TBL) is a teaching strategy that has gained popularity recently. Unlike conventional lectures, TBL is learner-centered and enhances learning effectiveness via team member participation [16]. Course designs comprise five stages: pre-class preparation, individual readiness assurance tests (IRATs), team readiness assurance tests (TRATs), immediate feedback/clarification, and clinical problem-solving activities [16, 17]. Learners use the pre-class preview to acquire memorized knowledge independently. During class time, they can conduct activities that emphasize applying knowledge and enhance critical thinking instead of reciting knowledge [18, 19]. Courses only require one main teacher who is sometimes assisted by teaching assistants; therefore, they can even be implemented in classes containing many students [16]. Many studies have demonstrated the effectiveness of TBL, showing its superiority over conventional face-to-face lecturing while also involving communication skills and abilities, critical thinking, and problem-solving skills [16, 20]. Aside from being applied to students, TBL has also been applied to educational training for clinical nurses [21]. Studies revealed that clinical nurses perceived increased learning quality and positive learning experiences with this learning method [22] and acquired knowledge and skills [23, 24].

Due to the coronavirus (COVID-19) pandemic, online learning has become the mainstream teaching strategy for continuing education among healthcare professionals. Online learning can deliver current information rapidly and flexibly. It can also take place at the learners’ pace, regardless of their geographical locations, and materials can be accessed at any time [25, 26].

However, a systematic review of factors affecting e-learning in health sciences education highlights key factors that may hinder this learning method: lack of equipment or users’ information technology skills, poor course structure, without pedagogic design, poor student engagement, and lack of teacher-student interaction [26].

Approximately 30–50% of the Taiwanese population has insufficient or limited HL [27]. Community healthcare providers (CHPs) play a crucial role as frontline providers to deliver healthcare information. A study showed that CHPs were unfamiliar with HL and lacked confidence in implementing HL practices. Thus, on-the-job training regarding HL literacy is needed [28].

Team-based learning (TBL) emphasizes communication and cooperation through small-group discussions among learners. This is an effective teaching strategy for cultivating CHPs’ HL competency. Studies revealed online TBL combining physical, online, and flipped classrooms is an effective learning method in health care education [29, 30]. At the moment of the COVID-19 pandemic, a well-structured online teaching strategy applying to on-the-job education for healthcare providers is needed. This study aimed to develop a hybrid online TBL model that can be implemented during on-the-job training of CHPs in Taiwan and to evaluate its effectiveness.

The findings are expected to provide a reference for current HL education and training.

## Methods

There are 374 community health centers in Taiwan which are important executive units of community health responsible for health promotion, disease prevention, and chronic disease management [31]. The HL training program has been piloted since 2019, and the data of this study are from the 2020 program.

### Study design

A single-group, pre-experimental, pretest–posttest design, and focus group interviews were conducted. Data on CHPs’ familiarity with HL, attitude toward HL, and confidence in implementing HL practices were collected before and after the experimental intervention, and the changes between the two time points were compared. Focus group interviews were used to collect and analyze the experience of research participants in the class, develop more in-depth and comprehensive contact with participants, and thus understand their learning and interpretation in the course participation process [32].

### Study setting and participants

The study sites were community health centers, divided according to their geographical distribution into four regions: northern Taiwan, central Taiwan, southern Taiwan, eastern Taiwan and offshore islands. Among the community health centers registered to participate in the course, 20 centers were sampled from each region, and all CHPs at each center who met the inclusion criteria were enrolled. The inclusion criteria were individuals aged 20 years or over, a full-time staff member in the community health center. The exclusion criteria were individuals employed as temporary workers or interns. A total of 104 CHPs participated before the course intervention, of whom 81 completed the course and the pre-and post-test questionnaires, with a completion rate of 77.88%.

On the other hand, the focus group interviews adopted purposive sampling to select participants who have completed the whole course and questionnaire. A total of 3 online focus group interviews with 6–7 participants each were held.

### Intervention

#### HL course

The course content of the intervention was developed regarding that of HL training for professionals [12, 14]. A six-week course combining the communication competencies of CHPs [33] and the practice of community HL interventions [34] with 90 min class once a week was designed. The course content covered four teaching modules: introduction to HL, oral communication skills, written communication skills and community HL intervention, and two case discussion sessions.

#### Online team-based learning

The first four teaching modules were implemented using the following steps of online TBL: pre-class preparation, readiness test, immediate feedback and clarification, and clinical problem-solving activities to strengthen the application of knowledge [16] (Additional file 1: Appendix 1). The formation of groups was based on health centers, with 20 groups and 4–6 people per group. The classes were held in their workplace, and one module was taught per week.

#### Pre-class preparation

Teaching videos were provided to participants to preview before class. Each video was approximately 14–20 min in length. One week before class, the videos were uploaded to the YouTube platform and the link was sent to the participants.

#### Individual readiness assurance test

Each module was designed with 5–12 multiple-choice questions. The IRAT was built on the Google Form platform. At the beginning of class, the participants were linked to the form and started answering.

#### Team readiness assurance test

Participants were provided with the same test questions as those in the IRAT and an Immediate Feedback Assessment Technique (IF-AT) card; subsequently, the TRAT was performed in teams after the IRAT. All team members were required to discuss and share the outcomes of their pre-class preparation and scratch out the correct answers on the IF-AT card per test item. If an incorrect answer was selected, the discussion continued until the correct answer was scratched out. Thus, the IF-AT card could reflect the answering process of each team.

#### Immediate feedback/ clarification

Explanations and discussions were conducted for questions with a poor correct response in the readiness tests, followed by a brief review of the essential points of the course.

#### Clinical problem-solving activities

This step covered four modules as well, as shown in Table 1. Classroom activities were designed for each module. Participants were required to conduct comprehensive application exercises on the knowledge learned through cooperation with their team members. Each team was required to report their implementation results, followed by interactions and discussions between different teams.

**Table 1:** Demographic information

Variable	Category	n	%
**Gender**	Male	8	9.9
	Female	73	90.1
**Occupation**	Nurse	59	72.8
	Other medical personnel	7	8.7
	Health administrators and others	15	18.5
**Age**	≤ 30 years	4	5.0
	31–50 years	65	80.2
	≥ 51 years	12	14.8
**Education**	College (5-year program)	16	19.8
	University and graduate school (Master)	65	80.2
**Location of service organization**	Northern Taiwan	20	24.6
	Central Taiwan	14	17.3
	Southern Taiwan	21	25.9
	Eastern Taiwan and offshore islands	26	32.1
**Working experience**	≤ 10 years	17	21.0
	11–20 years	39	48.1
	≥ 21 years	25	30.9
**Previous experience in HL training and education courses**	Never	30	37.0
	≤ 10 h	45	55.6
	≥ 11 h	6	7.4

#### Practical case discussion

**Fig. 1:**
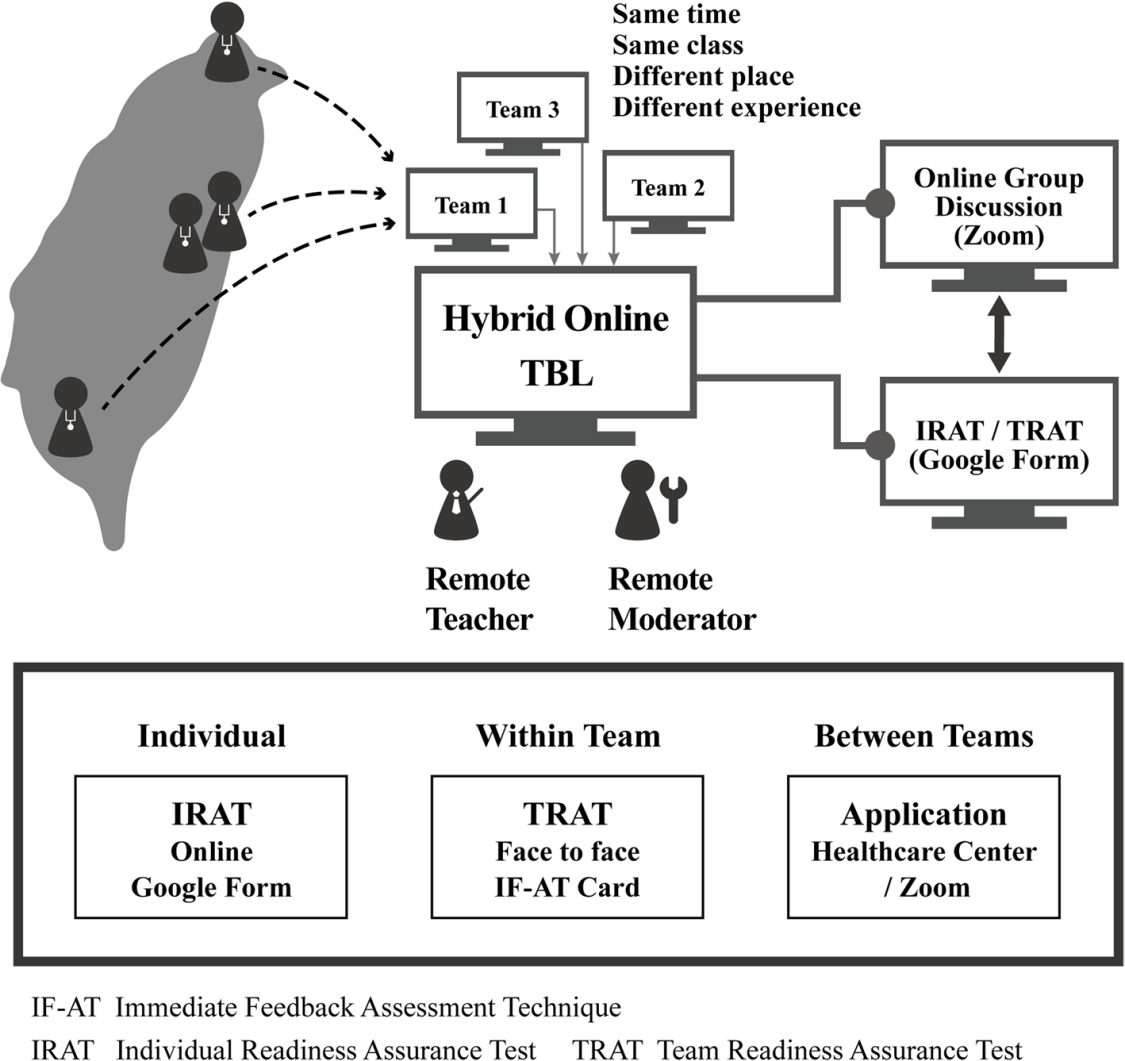
Hybrid online team-based learning for community healthcare providers

Our course design included two modules of practical case discussion, focusing on community elders’ communication and health centers’ practical experience in HL. Two experts were invited to share their practical experiences: (a) a nursing expert conducting health promotion programs for the elderly in the community for many years; (b) the director of a health center that had won the community HL organization award. Figure 1 displays the teaching strategy of the training program.

### Outcome variables and measurements

#### Familiarity with HL, attitude toward HL, and confidence in implementing HL practices

The research instrument published by Chang et al. for measuring CHPs’ familiarity with HL, attitude toward HL, and confidence in implementing HL practices were employed in this study [28]. This instrument was developed concerning the relevant literature while also accounting for the characteristics of community health work specific to Taiwan. The questionnaire includes 10 items on familiarity with HL, five items on the attitude toward HL, and eight items on confidence in implementing HL practices. Each item is self-rated by the participants on an 11-point scale, with 10 indicating “very familiar/agree/confident” and 0 denoting “very unfamiliar/disagree/not confident.” The content validity index for the applicability and clarity of the questionnaire was 0.97–1.0 and 1.0, respectively. The Cronbach’s Alpha was 0.97–0.98, indicating that the instrument has good reliability.

#### Course satisfaction

Course satisfaction was measured using an instrument developed by the researchers and comprises three parts: learning strategies (10 items), helpfulness of course modules to the implementation of HL practices (6 items), and overall feedback (4 items). The items were rated on a 5-point Likert scale, with a total of 20 items. Higher scores indicated greater satisfaction with the course.

#### Course experience

Focus group interviews were conducted using semi-structured interview guidelines to guide participants in sharing their experiences participating in this course. The topics covered included the method of delivery, learning process, and limitations.

### Data collection procedure

The quantitative research questionnaire was distributed electronically and instructions on filling in the online questionnaires. One week before the intervention, the link was sent by email to the online portal of each study site, which was then forwarded to the participants. Before the course intervention, each health center received a course kit, which contained operation instructions, sealed test papers, IF-AT cards, and other supplementary tools (such as a desktop camera with a microphone). The research assistant created online groups for each participating health center to provide a channel for consultations and problems solving during class. The course intervention lasted from July to August 2020 for six weeks. One week after the end of the course, the post-test questionnaires were distributed again.

Three focus group interviews were held after 1 week when the intervention course was completed.

The research assistant contacted research settings leaders to assist with participant recruitment and to provide information about focus groups. A moderator with experience in moderating focus group meetings was responsible for ensuring the smooth running of the discussion. At the beginning of the meeting, the moderator explained the purpose of the interview and asked for consent for audio and video recording. The moderator then used the semi-structured interview guidelines to lead the participants to discuss their learning experiences and feelings about the course. Each focus group session was approximately 60–90 min.

### Data analysis

Quantitative data were converted into Statistical Package for the Social Sciences (SPSS) data files, and descriptive statistics were performed. For qualitative data, the recorded audio files were transcribed verbatim. Two data analysts performed a content analysis to code the conceptual units line-by-line. The coded units were then summarized into categories and themes, which were named to represent the participants’ experiences.

### Research ethics

This study was reviewed by the medical institution’s research ethics committee. The IRB license number is IRB109-151-B.

## Results

### Demographic variables

A total of 81 CHPs completed the course and data collection. The data of the demographic variables are shown in Table 1. Among the participants, 90.1% were female, 72.8% were nurses, and 80.2% were aged 31–50 years, with a mean age of 43.06 ± 8.30 years. Further, 80.2% received college education or above, 79.0% had > 10 years of working experience, and 37.0% had never attended an HL course.

### Changes in familiarity with HL, attitude toward HL, and confidence in implementing HL practices

The pre-and post-test score changes are shown in Table 2. The mean score of familiarity with HL increased from 4.29 ± 1.76 to 6.92 ± 1.52 (t = 11.89, *p* < 0.001), and that of attitude toward HL increased from 7.39 ± 1.88 to 8.10 ± 1.44 (t = 2.98, *p* = 0.004), and that of confidence in implementing HL practices increased from 6.22 ± 1.48 to 7.61 ± 1.34 (t = 7.35, *p* < 0.001). All three outcome measures showed statistically significant differences before and after the intervention.

**Table 2:** Changes in familiarity with HL, attitude toward HL, and confidence in implementing HL practices

Item	Pre-test	Post-test	t	*p*-value
	*m* ± *SD*	*m* ± *SD*		
**Familiarity with HL**	4.29 ± 1.76	6.92 ± 1.52	11.89	.000
**Attitude toward HL**	7.39 ± 1.88	8.10 ± 1.44	2.98	.004
**Confidence in implementing HL practices**	6.22 ± 1.48	7.61 ± 1.34	7.35	.000

Note: scale 0–10 points

### Course satisfaction analysis

The mean score for participants’ satisfaction with the teaching strategies applied in this course was 4.07 ± 0.53 (the total score was 5 points), among which the flexibility of learning space scored the highest (4.26 ± 0.63). Further, the mean score for the helpfulness of the course modules in implementing HL practices was 4.13 ± 0.55, among which the introduction to the HL concept scored the highest (4.21 ± 0.56). Finally, the mean score for overall feedback on learning satisfaction was 4.06 ± 0.58, and the degree of agreement with conducting subsequent courses using the same method was 4.11 ± 0.63(Table 3).

**Table 3:** Analysis of satisfaction with the teaching methods and content

Item	*m* ± *SD*
**Teaching strategies**	4.07 ± .53
The preparatory videos increased my understanding of the course	4.14 ± .65
The IRATs increased learning motivation	4.01 ± .73
The TRATs increased mutual learning among group members	4.02 ± .65
The discussion activities enhanced the applicability of course content	4.06 ± .58
The learning time of the course was flexible	4.15 ± .65
The learning space of the course was flexible	4.26 ± .63
The course increased my interest in learning	4.01 ± .62
The course reduced learning stress	3.95 ± .84
The course ran smoothly	4.06 ± .60
The course content is easy to absorb	4.09 ± .62
**Helpfulness of the course modules to practice**	4.13 ± .55
Introduction to the HL concept	4.21 ± .56
Oral communication	4.14 ± .65
Written communication	4.11 ± .61
Community HL interventions	4.10 ± .60
Case discussion of communication with the elderly	4.15 ± .59
Case discussion of community HL interventions	4.05 ± .63
**Overall feedback**	4.06 ± .58
Familiarity with HL after the course	4.10 ± .56
Execution power of providing HL services after the course	3.89 ± .61
Overall, I am satisfied with the course	4.12 ± .53
The next course should adopt the same model	4.11 ± .63

Note: 5: Very helpful/familiar/agree; 4: Helpful/familiar/agree; 3: Neither helpful/familiar/agree nor unhelpful/unfamiliar/disagree; 2: Unhelpful/unfamiliar/disagree; 1: Very unhelpful/unfamiliar/disagree

### CHPs’ experience with a course participation

Three focus group interviews were conducted with 20 CHPs, among whom 95% (19) were community nurses and 5% (1) were health administrators. The mean age was 42.00 ± 6.78 years, the length of working experience was 19.11 ± 7.62 years, 20% (4) were health center supervisors, and 50% (10) had not attended a course on HL previously. Based on the content analysis of the interview data, 2 categories, 7 themes, and 13 subthemes were summarized (Table 4).

**Table 4:** Analysis of CHPs’ experience of course participation

Category	Theme	Subtheme
1. Experiences with teaching strategies	1. The distance learning course increased participation and accessibility	
	2. Series short course learning made learning easy	
	3. TBL was a new experience	3.1 The preparatory videos were helpful for learning 3.2 IRATs increase learning motivation 3.3 TRATs enhance the sharing of learning experiences 3.4 Team application activities enhance practical applications 3.5 TBL increased interactions between teams
	4. Case discussion provided experience	
	5. Challenges in learning	5.1 Video equipment and network problems 5.2 Unable to prepare before each class due to busy work schedule 5.3 Work-related interruptions during the course led to fragmented learning 5.4 Supervisors’ leadership eliminated all difficulties
2. Learning experiences in the HL course	6. The course content was systematic and complete	
	7. Review existing community health services and make improvements from the perspective of HL	7.1 Community health education activity 7.2 Written communication skills for HL 7.3 Community HL intervention 7.4 Health-literate healthcare environment

### Category 1 Experiences with teaching strategies

#### Theme 1 The distance learning course increased participation and accessibility

Many participants expressed that the distance learning course allowed them to study locally, reducing the transportation time, especially for CHPs living in remote areas or offshore islands. “*Since we live on offshore islands, it usually takes two days of return travel to attend classes. The distance learning course is efficient and does not take much time.*” *“It’s great to have classes at my own health center*.” Distance learning courses provide great convenience, while TBL conducted within health centers has also altered the predicament of “*only allowing a few delegates to attend,*” as in the past. Owing to the limited workforce and heavy workload, primary health centers often have to sacrifice the opportunity to participate in on-the-job training. Distance learning courses can be taught without leaving the health center, and TBL “*allows all members to participate*,” which increases the accessibility of on-the-job training in remote areas or offshore islands.

#### Theme 2 Series short course learning made learning easy

The participants pointed out that the on-the-job training courses they had previously participated in often lasted for many hours at a time. They thought that this course was different from the general training courses, as it was divided into six modules, with 90 min per week. The participants expressed that “*This course is divided into stages, so only a little content is received at a time, which is easy to absorb*.” Thus, a series of short learning courses made the course content easy to absorb.

#### Theme 3 TBL was a new experience

All the participants indicated that this was the first time they had taken part in TBL, and the following findings were obtained based on the participants’ experience:

### Subtheme 3.1 The preparatory videos were helpful for learning

The participants generally thought that the online preparatory videos could be viewed multiple times, without space and time constraints. *“You can watch the preparatory materials repeatedly, and I think they are very useful,” “… you can watch it even when taking the bus or MRT after work*.” The participants also expressed that “*pre-class preparation makes it easy to grasp the key points in class*.”

### Subtheme 3.2 IRATs increase learning motivation

A large amount of positive feedback was obtained for the pre-class tests conducted during TBL. Although the pre-class test placed pressure on the participants to prepare in advance, participants expressed that *“everyone is more nervous because there is a test before class.”* It also increased their learning motivation: “*As there is an exam, I will set aside some time to prepare for class no matter what; otherwise, it will be embarrassing*.” The test questions also provide a reminder of the key points in class. “*If you find that you have answered so many questions incorrectly, you will want to know more about the content that you have answered incorrectly.*” Students had a more profound impression of questions answered incorrectly, which improved their motivation for acquiring knowledge and increased their attention in class.

### Subtheme 3.3 TRATs enhance the sharing of learning experiences

In the TRATs, team members had to fill in the IF-AT card together, and if an answer was wrong, they were required to discuss it until the correct answer was selected. A participant stated, “*By answering together, I will discover key points that I had not noticed, but my colleagues had. By sharing with other team members, the key points of these courses can be quickly condensed together, and everyone can quickly absorb it.*” Through the group discussion, team members could examine deficiencies in their own learning and absorb the learning experiences of others.

### Subtheme 3.4 Team application activities enhance practical applications

Many participants shared that the skills learned in classroom activities, including oral and written communication, could be immediately applied to interactions with the public in community care activities while also rectifying their past communication methods. A participant stated, *“Sometimes we may accidentally use some technical terms that the elderly may not understand, but they don’t say so… after taking this class, I have a greater awareness. What we provide them is not necessarily what they need or the things they understand.*”

### Subtheme 3.5 TBL increased interactions between teams

Some participants had previously participated in distance learning courses, but most of their experiences were lecture-based courses that lacked interactions. “*In the past, most courses were one-way passive acceptance of lectures delivered by lecturers. Even in distance learning courses, we were limited to one person asking and one person answering at a time … This course added guidance by the lecturer and group discussions so that we (the same group) could share and brainstorm with each other.*” TBL involved interactions among students within the same group and the exchange of experiences among health centers in different regions. A participant stated, “*We can communicate with health centers in various counties and cities in such a short time. I think this is a commendable aspect of this course*.”

#### Theme 4 Case discussion provided experience

Case sharing and discussion provided learners with examples for emulating and learning, which can then be applied to improve their own practice. A participant pointed out the following: *“I really like the innovative practices of Datong District (Community Health Service Center). The part related to improving HL among the elderly is constructive for us when operating within the community. We want to use this approach to improve the seniors’ healthy meal activities that are currently being promoted.*”

#### Theme 5 Challenges in learning

In addition to the positive experience, the participants noted many challenges in learning related to the approach adopted in this course. The following four subthemes were summarized.

### Subtheme 5.1 Video equipment and network problems

The participants expressed occasional problems with a signal interruption during class, such as not hearing sounds or the microphone failing. “*Sometimes everything is fine when you’re listening to the teacher speaking but then find that there is no sound when it is your turn to speak.*” Thus, issues with network stability and software operation led to many problems.

### Subtheme 5.2 Unable to prepare before each class due to busy work schedule

Although the students knew that they needed to prepare before class, many participants also admitted that they could not finish their pre-class assignments before every class due to their busy work schedules. “*Work was hectic and tiring, so the pre-class preparation could not be completed every week*.”

### Subtheme 5.3 Work-related interruptions during the course led to fragmented learning

When the course took place on a workday, and there was official business to handle, the students often had to leave the classroom. The participants expressed that *“I can do nothing about it. If people come, I have to communicate with them*, which fragmented the learning process.” A participant pointed out, *“it’s a pity that my colleagues gave up halfway and didn’t finish the whole course because they missed some classes due to business or meetings*.” As this course lasted for six weeks and was held on weekdays, it was not easy for participants to attend every class.

### Subtheme5.4 Supervisors’ leadership eliminated all difficulties

The focus group interviews were attended by four supervisors, who noted that to improve course participation and learning outcomes, some measures should be implemented to assist their colleagues with learning. A participant pointed out, “*In particular, I would avoid arranging business trips for my colleagues as far as possible so that everyone can participate. If some colleagues are absent from certain classes, I would ask them to watch the videos and practice the questions for the class (readiness test).*” Thus, the leadership provided by the supervisors could increase their colleagues’ participation.

### Category 2 Learning experiences in the HL course

#### Theme 6 The course content was systematic and complete

HL is a new concept for some participants. When the health center announced the requirement to attend the HL course, the participants expressed that their first response was as follows: “*What is HL*?” One participant said: “*I have only taken one-off classes before, and my impression of HL is that it should be ‘easy to understand’, but I don’t know how to use it.*” This course included a series of classes, which participants considered “*very detaile*d” and “*very complete*.” During the classes, they felt that “*HL is closely related to the work that [they] are conducting*” and *“not only those who undertake HL need to learn it, but everyone (professionals) should learn it*.”

#### Theme 7 review existing community health services and make improvements from the perspective of HL

During the course, the participants could employ the concept of HL to examine the work for which they were responsible.

### Subtheme 7.1 community health education activity

The participants indicated that they often needed to conduct health education when providing community health services. This course involved oral and written communication skills, which allowed them to constantly remind themselves of the critical points that require attention when providing public health education. The participants pointed out that health education activities in the community were often under the pressure of reaching the target number in the past, with many different types of target audiences. Thus, leaflets, posters, and other standard educational materials were often used, which the participants felt “*was not well-received by the public*.” This course “*led to gradual changes in*” their health education activities. CHPs would reflect as follows: “i*f we express something in this way, will the public (literacy level) accept it or not”; “Now we try to meet the needs of the public (literacy level) and then provide them with the appropriate teaching materials*.” Regarding the topics covered in health education materials, the participants pointed out that in the past, the health education materials *“were to meet their own (professionals) goals, instead of understanding what the public in this community actually wants (health information). It forced them (the public) to listen to such topics*.” The HL course enabled participants to pay attention to the HL needs of the public when conducting health education activities.

### Subtheme 7.2 written communication skills for HL

This course introduced the design principles of health educational materials and conducted evaluation exercises. A participant noted that “*Leaflets are needed for publicity in the community. Previously, we ignored whether people had a problem understanding them. Now, we know that there are criteria (criteria for the suitability of educational materials), so it is easier for me to evaluate whether [the leaflets] meet these criteria*…”.

### Subtheme 7.3 community HL intervention

Community HL intervention units were introduced into the planning practice of community activities with the concept of empowerment, and the participants thought that this was “very practical.” “*This exercise helped everyone clarify many misunderstandings. In terms of public participation, in the past, everyone thought that it only involved having a certain number of people participating in these activities but neglected to ask the public’s opinions when planning and understand what information the public needed.*”

### Subtheme 7.4 health-literate healthcare environment

Regarding the content of HL-friendly institutions and navigation, the participants expressed that *“such learning is instrumental*.” The participants pointed out that these indicators could be used to examine their own health centers, which also echoed the evaluation of health centers. “*When people come in (health centers), they will ask where the dentistry department is. This makes me think that when I have to compile the Elderly-Friendly Program next year, I can use the funds to make a board (direction sign). That is, I will come up with other ideas. After this course, I have a clearer concept of why I should do this.*”

## Discussion

This study conducted on-the-job education and training on HL among CHPs in Taiwan through hybrid online TBL. The course reached all administrative regions in Taiwan and the offshore islands. The application of digital technology in online learning has enabled learners to study across different regions without constraints in time and space, thereby expanding their learning opportunities [25]. Internet usage is widespread among Taiwanese people, with an Internet access rate of 85.6%. Thus, Taiwan is considered a digital society [35], and the promotion of online learning is now even more feasible. CHPs are often required to undertake a heavy workload [36], and on-the-job training is another burden they must shoulder. In this study, online learning was applied in the workplace. Our findings demonstrated that “flexible learning time and space” was the item with the highest satisfaction in this course. According to the qualitative analysis, participants felt that online learning could overcome the inconvenience of traffic and time and that studying in the workplace was convenient. The findings show that online learning is effective and can be applied in on-the-job education and training for healthcare providers in Taiwan.

Online learning can increase the accessibility of on-the-job education and training, increase the participation rate of personnel, and hence achieve the goals of on-the-job education. However, a research article has noted that online learning may be hindered by equipment problems or users’ unfamiliarity with digital equipment [26]. This study provided operation instructions and digital equipment before the course. Nevertheless, interviewees still mentioned problems with disruptions in learning caused by network interruption or lack of sound. Thus, the stability of network transmission and learners’ digital literacy may still influence factors that need to be considered when further promoting online courses.

The professional competencies of HL include knowledge, attitude, and skills [10, 12, 13], while the course content covered vast memory-based and comprehension-based knowledge. By adopting TBL in this study, memory-based content was pre-recorded and provided to participants for independent learning; face-to-face classroom time focused more on establishing attitudes and learning skills. HL is still a new concept in Taiwan, and most CHPs are still unfamiliar with it, showing poor confidence in implementing HL practices [28]. Therefore, in addition to teaching through TBL, this course included two modules to share practical experience and strengthen the demonstration of practical implementation. The results showed that CHPs’ familiarity with HL, attitude toward HL, and confidence in implementing HL practices improved significantly after the course. Furthermore, based on qualitative data analysis, CHPs could apply the acquired skills in their work. They exhibited the ability to reflect and change when providing services, including community health education activities, selection of written health education materials, community HL interventions, and navigation through the institutional environment. The survey on learning satisfaction showed a high degree of satisfaction with the course strategies and learning outcomes. This study shows that TBL is an effective strategy for teaching HL in CHPs. Students can learn knowledge related to HL through self-learning before class, and learn skills through classroom learning activities [18, 19]. This strategy can meet the need for the cultivation of HL-related competencies of CHPs, including knowledge, attitude, and skills [10, 12, 13].

TBL created opportunities for students to interact with each other. Ideally, learning teams should be formed by team members from different backgrounds as far as possible to enhance diversity [16]. In this study, each health center formed one team. Although this did not fully conform to the principle of TBL grouping, qualitative data analysis showed that the participants also had positive learning experiences from the interactions among team members and different teams. This is consistent with the findings of Considine et al. that nurses felt that TBL allowed their team to provide immediate feedback and that other people’s ideas made them aware of their shortcomings and enabled them to learn about different viewpoints, thereby improving their knowledge and skills [22].

Currently, on-the-job HL education and training are mainly for professionals in medical institutions, such as residents, specialist nurses, and social workers [11, 37, 38]. Conversely, few HL courses are focused on CHPs serving the community. In this study, we designed an HL course for CHPs, which included HL-related knowledge, oral and written communication skills for HL, the promotion of community HL interventions, and discussion on HL among the elderly. We aimed to ensure that CHPs cooperate with community partners, employ methods and approaches to disseminate public information and data, and apply theories or models to convey information and change behaviors [33]. The satisfaction survey showed that the course was helpful, while qualitative data revealed that it enabled CHPs to examine and improve community health services from the perspective of HL.

## Conclusions

Medical knowledge and skills are changing with each passing day, and healthcare providers should receive on-the-job training constantly to meet the practical needs of their work. HL is one of them. However, due to heavy workloads and time constraints, on-the-job training is another burden healthcare providers must shoulder. This study developed an HL course suitable for CHPs, and online learning was used to increase participants’ accessibility. TBL and case discussion provided opportunities for exercises and discussions on practical skills while also increasing learning autonomy and interactions. Our research results showed that this hybrid online TBL model was effective and provided positive experiences for CHPs. Therefore, we have proposed a feasible on-the-job training model in this study that incorporates innovative teaching through online learning and can serve as a reference for the on-the-job training of various healthcare workers.

Limitations.

There was no control group in our research design, so we cannot confidently confirm whether the changes over time were due to participation or to other factors.

On the other hand, the outcome indicators of this study only discuss the improvement of the HL-related competencies of healthcare providers and do not discuss the indicators related to healthcare consumers. It is recommended that the outcome variables of healthcare consumers be included in a future study, which could better reflect the effect of HL education and training of healthcare providers.

## Supplementary Information

**Additional file 1.**

## Data Availability

The data that support the findings of this study are available from Health Promotion Administration, Ministry of Health and Welfare. However, restrictions apply to the availability of these data, which were used under license for this study, and are therefore not publicly available. Nevertheless, data are available from the corresponding author, Mei-Chuan Chang, upon reasonable request and with permission of the Health Promotion Administration, Ministry of Health and Welfare.

## Abbreviations

HL: Health literacy
CHPs: Community health providers
TBL: Team-based learning

## References

[1] Nielsen-Bohlman  L,  Panzer  A, Kindig  D (2004). Health literacy: a prescription to end confusion. Washington D.C.: The National Academies Press.

[2] Berkman ND, Sheridan SL, Donahue KE, Halpern DJ, Crotty K (2011). Low health literacy and health outcomes: An updated systematic review. Ann Intern Med.

[3] Abrams MA, Kurtz-Rossi S, Riffenburgh A, Savage BA. Building Health Literate Organizations: A Guidebook to Achieving Organizational Change. 2014. Available from http://www.HealthLiterateOrganization.org. Accessed 31 Mar 2022.

[4] Coleman C (2011). Teaching health care professionals about health literacy: A review of the literature. Nurs Outlook.

[5] Loan LA, Parnell TA, Stichler JF, Boyle DK, Allen P, VanFosson CA, Barton AJ (2018). Call for action: nurses must play a critical role to enhance health literacy. Nurs Outlook.

[6] Cafiero M (2013). Nurse practitioners' knowledge, experience, and intention to use health literacy strategies in clinical practice. J Health Commun.

[7] Macabasco-O'Connell A, Fry-Bowers EK (2011). Knowledge and perceptions of health literacy among nursing professionals. J Health Commun.

[8] Güner MD, Ekmekci PE (2019). A survey study evaluating and comparing the health literacy knowledge and communication skills used by nurses and physicians. Inquiry.

[9] Nesari M, Olson JK, Nasrabadi AN, Norris C (2019). Registered nurses' knowledge of and experience with health literacy. Health Lit Res Pract.

[10] Coleman CA, Hudson S, Maine LL (2013). Health literacy practices and educational competencies for health professionals: A consensus study. J Health Commun.

[11] Green JA, Gonzaga AM, Cohen ED, Spagnoletti CL (2014). Addressing health literacy through clear health communication: A training program for internal medicine residents. Patient Educ Couns.

[12] Kaper MS, Sixsmith J, Koot JA, Meijering LB, van Twillert S, Giammarchi C (2018). Developing and pilot testing a comprehensive health literacy communication training for health professionals in three European countries. Patient Educ Couns.

[13] Toronto CE (2016). Health literacy competencies for registered nurses: An e-Delphi study. J Contin Educ Nurs.

[14] Mackert M, Ball J, Lopez N (2011). Health literacy awareness training for healthcare workers: Improving knowledge and intentions to use clear communication techniques. Patient Educ Couns.

[15] McCleary-Jones V (2016). A systematic review of the literature on health literacy in nursing education. Nurse Educ.

[16] Cho YH, Kweon YR (2017). Effects of team-based learning on communication competence for undergraduate nursing students. J. Korean Acad. Psychiatr. Mental Health Nurs..

[17] Burgess A, van Diggele C, Roberts C, Mellis C (2020). Team-based learning: Design, facilitation and participation. BMC Medical Educ.

[18] Corbridge SJ, Corbridge T, Tiffen J, Carlucci M (2013). Implementing team-based learning in a nurse practitioner curriculum. Nurse Educ.

[19] Reimschisel T, Herring AL, Huang J, Minor TJ (2017). A systematic review of the published literature on team-based learning in health professions education. Med Teach.

[20] Oldland E, Currey J, Considine J, Allen J (2017). Nurses’ perceptions of the impact of team-based learning participation on learning style, team behaviours and clinical performance: An exploration of written reflections. Nurse Educ Pract.

[21] Considine J, Berry D, Allen J, Hewitt N, Oldland E, Sprogis SK, Currey J (2021). Team-based learning in nursing education: A scoping review. J Clin Nurs.

[22] Considine J, Currey J, Payne R, Williamson S (2014). Participant evaluation of team-based learning using one-off teams in a hospital setting. Australas Emerg Nurs J.

[23] Kim S, Kim CG (2020). Effects of an electrocardiography training program: Team-based learning for early-stage intensive care unit nurses. J Contin Educ Nurs.

[24] Seo Y, Roh YS (2020). Effects of pressure ulcer prevention training among nurses in long-term care hospitals. Nurse Educ Today.

[25] Mittelmeier J, Rienties B, Rogaten J, Gunter A, Raghuram P (2019). Internationalization at a distance and at Home: Academic and social adjustment in a South African distance learning context. Int J Intercult Relat.

[26] Regmi K, Jones L (2020). A systematic review of the factors–enablers and barriers–affecting e-learning in health sciences education. BMC Medical Educ.

[27] Wei MH, Wang YW, Chang MC, Hsieh JG (2017). Development of Mandarin Multidimensional Health Literacy Questionnaire (MMHLQ). Taiwan J Public Health.

[28] Chang MC, Hsieh JG, Wei MH, Tsai CH, Yu JH, Wang YW (2021). Familiarity, attitude, and confidence of health literacy practice among community healthcare providers in Taiwan. Int J Environ Res Public Health.

[29] Franklin AS, Markowsky S, De Leo J, Normann S, Black E. Using team-based learning to teach a hybrid pharmacokinetics course online and in class. Am. J. Pharm. Educ. 2016; 25;80(10).Available from: https://www.ajpe.org/content/ajpe/80/10/171.full.pdf. Accessed 1 Apr 2022. 10.5688/ajpe8010171 PMC5289727 28179720

[30] Yu CH. Hybrid teaching mode including physical, online, and flipped classroom learning for dental education in Taiwan. J. Dent. Sci. 2022;17(1):624. Available from: https://www.ncbi.nlm.nih.gov/pmc/articles/PMC8740369/pdf/main.pdf . Accessed 1 Apr 2022. 10.1016/j.jds.2021.09.026 PMC8740369 35028106

[31] Health Promotion Administration. Annual statistical report from public health centers in Taiwan. 2018. Available from: https://www.hpa.gov.tw/Pages/Detail.aspx?nodeid=582&pid=11009. Accessed 31 Mar 2022.

[32] Song YT, Pan PY (2010). Application of mixed research in educational research. J Educ Sci Res.

[33] The Council on Linkages Between Academia and Public Health Practice. Core Competencies for Public Health Professionals. 2014. Available from: phf.org/core-competencies. Accessed 31 Mar 2022.

[34] Baur C, Martinez LM, Tchangalova N. Rubin D. A review and report of community-based health literacy interventions. Presentation and paper at Roundtable on Health Literacy, The National Academies of Sciences, Engineering, and Medicine, Washington, D.C. 2017. Available from http://www.nationalacademies.org/hmd/Activities/ PublicHealth/HealthLiteracy/2017-JUL-19.aspx Accessed 31 Mar 2022.

[35] Taiwan Internet Report. *2019 *Taiwan Internet Report. 2019. Available at: https://report.twnic.tw/2019/assets/download/TWNIC_TaiwanInternetReport_2019_CH.pdf Accessed 31 Mar 2022.

[36] Chen DY, Wu SW, Chen YC, Wang GX (2019). Factors associated with public health nurses’ burnout from the perspective of job demands-resources model: Public service motivation as a moderator. Taiwan J Public Health.

[37] Coleman CA, Nguyen NT, Garvin R, Sou C, Carney PA (2016). Health literacy teaching in US family medicine residency programs: A national survey. J Health Commun.

[38] Coleman CA, Fromer A (2015). A health literacy training intervention for physicians and other health professionals. Fam Med.

