# Supplementary material for: Health literacy training program for community healthcare providers using hybrid online team-based learning in Taiwan
Source: BMC Med Educ. 2022 Jul 27;22:576. doi: 10.1186/s12909-022-03646-7 (PMC9327261; doi:10.1186/s12909-022-03646-7)
Supplement: Supplementary file 1 — Additional file 1. [file 12909_2022_3646_MOESM1_ESM.docx]

Appendix 1 Teaching modules, content, and clinical problem-solving activities

| Teaching modules | Content | Clinical problem-solving activities |
| --- | --- | --- |
| Introduction to HL | The definitions of HL  The prevalence of inadequate HL and health outcomes  HL universal precaution approach  Health literate organization and navigation | Association with community HL issues  Description: Each group raised issues related to HL in community health services |
| Oral communication skills | The characteristics of people with low HL  Oral communication skills (chunk and check, teach back)  Encouraging patients to ask questions | Oral communication by finding fault together  Description: Each group watched a video about the communication process between a doctor and a diabetic elderly individual and then identified features of poor communication together and corrected them |
| Written communication skills | The criteria of health literate written educational materials | Evaluation of written health educational materials  Description: Each group analyzed the same health education leaflet using the criteria for health literate written educational materials and discussed it |
| Community HL intervention | How CHPs build health literate healthcare centers  How CHPs plan community-based HL programs | Community HL interventions planning exercise  Description: The group worked together to complete HL interventions for the same health issue and discussed it |
